# PERson-centredness in Hypertension management using Information Technology: a randomized controlled trial in primary care

**DOI:** 10.1097/HJH.0000000000003322

**Published:** 2022-11-18

**Authors:** Ulrika Andersson, Peter M. Nilsson, Karin Kjellgren, Mikael Hoffmann, André Wennersten, Patrik Midlöv

**Affiliations:** a Center for Primary Healthcare Research, Department of Clinical Sciences Malmö, Lund University; b Department of Clinical Sciences Malmö, Lund University, Malmö; c University of Gothenburg Centre for Person-Centred Care, University of Gothenburg, Gothenburg; d Department of Health, Medicine and Caring Sciences, Linköping University, Linköping; e Clinical Studies Sweden – Forum South, Skåne University Healthcare, Lund, Sweden

**Keywords:** blood pressure, digital intervention, E-health, hypertension, person-centred care, primary healthcare

## Abstract

To increase the proportion of individuals with hypertension obtaining a blood pressure (BP) of less than 140/90 mmHg by improving the management of hypertension in daily life from a person-centred perspective.

**Methods:**

In this unblinded randomized controlled trial, we tested an interactive web-based self-management system for hypertension. A total of 949 patients with hypertension from 31 primary healthcare centres (PHCCs) in Sweden were randomized 1 : 1 to either the intervention or usual care group. The intervention included daily measurement – via the participant's mobile phone – of BP and pulse and reports of well being, symptoms, lifestyle, medication intake and side effects for eight consecutive weeks. It also included reminders and optional motivational messages. The primary outcome was the proportion of participants obtaining BP of less than 140/90 mmHg at 8 weeks and 12 months. Significance was tested by Pearson's chi^2^-test.

**Results:**

A total of 862 patients completed the trial, 442 in the intervention group and 420 in the control group. The primary outcome (BP <140/90 mmHg) at 8 weeks was achieved by 48.8% in the intervention group and 39.9% in the control group (*P* = 0.006). At 12 months, 47.1% (intervention) and 41.0% (control group) had a BP less than 140/90 mmHg (*P* = 0.071).

**Conclusion:**

The proportion of participants with a controlled BP of less than 140/90 mmHg increased after using the interactive system for self-management of hypertension for 8 weeks compared with usual care. Although the trend continued, there was no significant difference after 12 months. The results indicate that the effect of the intervention is significant, but the long-term effect is uncertain.

**Trial registration:**

The study was registered with ClinicalTrials.gov (NCT03554382).

## INTRODUCTION

### Background

E-health solutions as part of emerging information and communication technologies (ICT) have been recognized as important tools to improve outcomes in the management of hypertension [1]. Several factors have been identified as central for improved blood pressure (BP) control. In particular, low patient adherence to treatment – intentional or unintentional – and physician inertia to adjust treatment, when appropriate, are in focus for patients diagnosed with hypertension [2]. E-health solutions have the potential to address these factors and reach many people as mobile phones and internet access are almost ubiquitous.

The problem of patients’ low adherence to treatment can be addressed by including reminders to take medication and personalized education [3]. E-health interventions often include self-monitoring of BP, which is increasingly recommended, as research indicates that when combined with education and counselling, it positively affects adherence and BP levels [4–6].

E-health interventions can also play an important role in changing interactions between patients and healthcare professionals [3,7]. The traditional role of the patient as a passive recipient of care is outdated and not compatible with the modern healthcare system. When the patient can gather health information and gain an understanding of the interplay between BP values, lifestyle, symptoms and treatment, the balance in the healthcare relationship shifts [8,9]. This method supports a person-centred approach, where the patient's beliefs, experiences and resources are recognized and emphasized. A shared decision-making process is recommended to improve adherence and BP control [10,11].

Home BP monitoring (HBPM) in hypertension treatment is increasingly recommended. It may promote person-centred care as it requires active participation from the patient [12]. Furthermore, interventions with self-monitoring of BP offer an opportunity for prescribing physicians to remotely monitor the patient's BP levels in an easily accessible way, thus potentially addressing the problem of therapeutic inertia [1].

Although there seems to be a lot to gain by using e-health interventions in hypertension management, there are issues that need to be resolved before the implementation of such services in clinical care. It is of vital importance that patient data is handled securely and the intervention needs to be scientifically validated before implementation [1,6]. Several smaller studies and pilot projects have tested different digital or mobile phone tools aimed at improving hypertension self-management and BP control [13–15]. The results are promising, although further research and larger trials of longer duration are required, especially in primary care [3,16–19].

### Objectives

The primary aim of the trial was to study the effect of a person-centred approach supported by e-health technology on the proportion of individuals being treated for hypertension obtaining a BP goal of less than 140/90 mmHg, by improving the management of hypertension in daily life.

## METHODS

### Study design and participants

PERson-centredness in Hypertension management using Information Technology (PERHIT) was an unblinded multicentre randomized controlled trial where an interactive web-based communication system, which used the patient's mobile phone for self-management of hypertension, was tested by patients and professionals in primary care in four healthcare regions in southern Sweden. It was previously described in the study protocol [20].

Invitation letters were sent out to the unit heads of all eligible primary healthcare centres (PHCCs) in the four regions. Those who responded and were interested in participating received further information. An information meeting was held at the PHCC, with nurses and physicians from the centre participating. At the meeting, the nurses and physicians received information and instructions on how to use the web-based interactive system. Participating PHCCs received financial compensation for each included patient and time spent taking part in the study. The participating patients did not receive any financial compensation.

Nurses and physicians at the participating PHCCs identified potentially eligible patients [20] and then informed them about the study. Information about the study was also available in the waiting rooms at the PHCCs and the patients could initiate their participation themselves. All patients who agreed to participate signed a consent document.

Inclusion criteria were Swedish-speaking adult patients with a diagnosis of hypertension and treatment with at least one antihypertensive drug. The actual blood pressure level at inclusion was not set as inclusion criteria, depending on common variations in blood pressure during the office measurement. Exclusion criteria were secondary hypertension, terminal illness, pregnancy-induced hypertension, cognitive impairment, impaired vision (not able to read messages on the mobile phone) and psychotic disorder.

The technology in the trial was designed to be easy to use and to not require any previous experience of using similar systems, thus not excluding patients with limited experience of technology use [20] (Excluded patients, see Supplementary Table).

After inclusion, the participants took part in a baseline assessment, which included measuring office BP (mmHg) and pulse (beats/min), height (m) and weight (kg), blood tests (cholesterol, creatinine, HbA1c and cystatin C) and answering questionnaires. After baseline assessment, the patients were randomized to the intervention or control group. The patients in the control group received care as usual and did not receive a BP monitor.

All participating patients were booked for follow-up visits at 8 weeks and 12 months. At these visits, the patients answered questionnaires, office BP and pulse were measured, and blood samples were taken [20].

### Intervention

**FIGURE 1:**
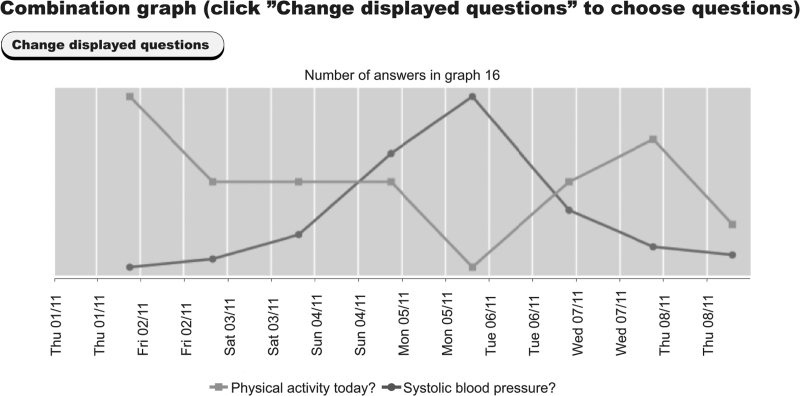
Example of an authentic graph displaying SBP in relation to physical activity. Data from [7]. The patient can choose, which data they want to be presented, and thereby see how, for example, physical activity, stress and medication intake affects their blood pressure.

A set of questions adopted for patients with hypertension for use in the web-based self-management support system was developed and evaluated by part of the research team in a pilot study with focus groups and individual interviews together with patients and healthcare professionals [8,21,22]. The web-based communication system Circadian Questions (CQ) was developed by Circadian Questions AB, Sweden and is referred to subsequently in this manuscript as ‘the system’ [7,20,22]. In short, the interventions consisted of three parts:

1. After randomization to the intervention group, the participants were instructed by their nurse or physician on how to measure their BP and how to use the system at home. They received a BP monitor (Microlife BP A6 BT; Microlife, Widnau, Switzerland) with a written manual and were informed about supporting video films on the study home page. Participants were encouraged to receive motivational messages for healthy habits, which were selected together with the healthcare professional. Reminders were adjusted according to the patient's preferences.
2. Every evening for eight consecutive weeks, the patients received a reminder via a text message sent to their mobile phone to measure and report their BP, pulse, medication intake, stress level, well being, physical activity, symptoms and side effects into the system. They received weekly motivational messages if they chose to receive them. Patients and professionals had access to a login-secured web portal for reported values that were visualized in graphs throughout the intervention period (Fig. 1).
3. After 8 weeks, the participants came back for a follow-up consultation with their nurse or physician at the PHCC. Patients and healthcare professionals were encouraged to use the graphs representing the patient's reported values as a basis for discussion.

### Blood pressure measurement

Office BP was chosen as the outcome as it is commonly used in clinical studies to evaluate the effect of a BP intervention. Office BP was measured at the PHCC by the patient's nurse or physician at baseline, at 8 weeks and at 12 months. Instructions for standardized BP measurement were given to the healthcare professionals at the local start-up meeting for the trial and via a website to ensure systematic BP measurements. After 5 min of rest, the patient's BP was measured in the upper arm with them in a sitting position. A validated electronic BP monitor (Microlife BP A6 BT) was used and the mean value of three consecutive measures was displayed and manually recorded in the electronic case report form (eCRF). The patients in the intervention group brought the same type of monitor home with them and used it for daily measurements during the intervention.

According to current European guidelines [23], an uncontrolled BP is defined as a mean office SBP of at least 140 mmHg and/or DBP of at least 90 mmHg.

Titration of antihypertensive drugs was not specified in the study instructions. If BP values were high or too low, the prescribing physician could modify the patient's treatment at any time during the intervention.

### Randomization

Patients were randomized 1 : 1 to the intervention or control group after baseline assessments. Block randomization was used to reduce the risk of bias and to ensure balance in the allocation of patients between sites. An independent statistician created the randomization table in the eCRF. The members of the research team did not have access to the randomization module. At each site, a qualified and independent monitor oversaw the data and study process.

### Data analysis

A power calculation was performed before the trial was initiated. After consulting previous literature on hypertension self-management interventions compared with usual care, an average difference in the decrease in SBP of 5.5 mmHg was suggested between baseline and after 12 months. Assuming a mean difference of 5 mmHg, a standard deviation of 20 mmHg in both groups, and a 20% drop-out, 423 patients in each group were required for 90% statistical power, at the 5% significance level.

As stated in the study protocol [20], the primary outcome was to calculate the proportion of patients achieving a BP goal of less than 140/90 mmHg at 8 weeks and 12 months in the intervention and the control group, respectively. Office BP was used. Analyses were done according to intention-to-treat and significance was tested by Pearson's chi^2^-test.

IBM SPSS statistics 27 (IBM Corp. Released 2020. IBM SPSS Statistics for Windows, Version 27.0, IBM Corp, Armonk, New York, USA) was used for all statistical analyses.

### Ethical considerations

The PERHIT-project was approved by the Regional Ethics Review Board in Lund (2017/311 and 2019/00036). Self-monitoring of BP at home could potentially stir feelings of anxiety or result in obsessional measuring. It was also possible that participants felt controlled or supervised during the intervention. However, the potential benefits of using the system were judged to exceed these potential adverse effects. Changes in vital signs were monitored at the local PHCC according to clinical routines.

The study was registered with ClinicalTrials.gov (NCT03554382).

The CONSORT checklist was followed to ensure scientific rigour in reporting the trial.

## RESULTS

### Participants

**FIGURE 2:**
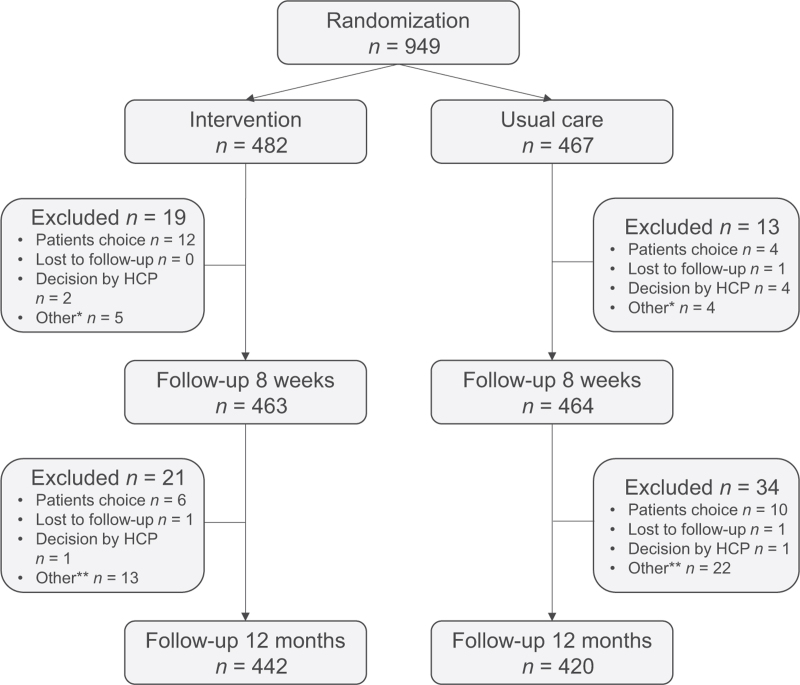
Flowchart of the PERHIT-trial. HCP, Healthcare professional. ^∗^Other reasons for exclusion: error in randomization, hospitalized, bad internet connection, mobile phone not working. ^∗∗^Other reasons for exclusion: PHCC did not complete the study, deceased, not visiting PHCC because of pandemic, were not called, unknown, spouse deceased, not been able to come to PHCC, illness. PHCC, primary healthcare centre.

In total, 949 patients with hypertension from 31 PHCCs were included in the trial. Eight hundred and sixty-two patients completed all the steps of the trial; 442 in the intervention group and 420 in the control group (Fig. 2). Participants were included between 24 October 2018, and 29 November 2019, and were followed for 1 year.

The characteristics of the participants are described in Table 1. The intervention and the control group were well matched regarding age, baseline BP, BMI, kidney function and cholesterol level. Men were predominant in both groups. Most participants stated that they were born in Sweden; only 26 participants were originally from other countries (12 in the intervention and 14 in the control group). About 50% of the participants were employed.

**TABLE 1:** Baseline characteristics of study participants in the intervention and control group

Characteristics	Total population (*n* = 949)	Intervention group (*n* = 482)	Control group (*n* = 467)
Age (years), mean (SD, range)	62.9 (9.9, 25–92)	62.8 (9.8, 25–85)	63.0 (10.0, 33–92)
Sex, women (%)	407 (42.9)	199 (41.3)	208 (44.5)
SBP (mmHg), mean (SD)	144.8 (16.7)	144.1 (16.6)	145.6 (16.9)
DBP (mmHg), mean (SD)	85.2 (10.0)	84.8 (9.2)	85.7 (10.7)
BMI (kg/m^2^), mean (SD, range)	28.9 (4.5, 17.6–47.4)	28.8 (4.3, 17.7–45.2)	28.9 (4.7, 17.6–47.4)
eGFR (ml/min/1.73 m^2^), mean (SD)	77.3 (25.4)	78.6 (33.5)	76.2 (14.0)
Total cholesterol (mmol/l), mean (SD)	5.0 (1.1)	5.0 (1.0)	5.1 (1.1)
Duration of hypertension (years), mean (SD)	10.4 (10.2)	9.6 (8.8)	11.1 (11.4)
Baseline number of antihypertensive drugs (median, interquartile range)	1 (1-2)	1.5 (1-2)	1 (1-2)
Diabetes [*n* (%)]	115 (12.1)	60 (12.6)	55 (11.9)
Current smoker [*n* (%)]	47 (5.0)	23 (4.8)	24 (5.1)
Previous smoker [*n* (%)]	240 (25.3)	123 (25.5)	117 (25.1)
Marital status [*n* (%)]			
Married or cohabiting	749 (78.9)	381 (79.0)	368 (78.8)
Unmarried or widowed	164 (17.3)	79 (16.8)	85 (18.4)
Other	18 (1.9)	10 (2.1)	8 (1.7)
Education level [*n* (%)]			
Up to high school	217 (22.9)	102 (21.8)	115 (25.0)
High school	430 (45.3)	216 (46.2)	214 (46.5)
University	281 (29.6)	150 (32.1)	131 (28.5)
Occupation [*n* (%] several occupations possible)			
Working	486 (51.2)	245 (50.8)	241 (51.6)
Retired	461 (48.6)	236 (49.0)	225 (48.2)
Other (e.g. student, unemployed)	22 (2.3)	13 (2.7)	9 (1.9)

Some percentages might not add to 100% because of rounding or missing values. eGFR, estimated glomerular filtration rate; SD, standard deviation.

### Blood pressure

At baseline, 35.5% of the participants in the intervention group and 35.3% in the control group had a BP of less than 140/90 mmHg. After using the system for 8 weeks, 48.8% of the participants in the intervention group had a BP of less than 140/90 mmHg, which was significantly better than in the control group, where 39.9% had a BP of less than 140/90 mmHg (*P* = 0.006). At 12 months, 47.1% in the intervention group had reached the target versus 41% in the control group but the difference was not statistically significant (*P* = 0.071) (Table 2). The improved BP consisted mainly of an improved SBP control.

**TABLE 2:** Comparison of controlled and uncontrolled blood pressure in the intervention and control group

	Visit	Intervention [*n* (%)]	Control [*n* (%)]	*P* value
BP <140/90 mmHg	Baseline	171/482 (35.5)	165/467 (35.3)	0.963
	8 weeks	226/463 (48.8)	181/454 (39.9)	**0.006**
	12 months	208/442 (47.1)	172/420 (41.0)	0.071
SBP <140 mmHg	Baseline	191/482 (39.6)	182/467 (39.0)	0.837
	8 weeks	254/463 (54.9)	198/454 (43.6)	**<0.001**
	12 months	225/442 (50.9)	195/420 (46.4)	0.189
DBP <90 mmHg	Baseline	331/482 (68.7)	299/467 (64.0)	0.130
	8 weeks	331/463 (71.5)	314/454 (69.2)	0.440
	12 months	325/442 (73.5)	299/420 (71.2)	0.443

BP, blood pressure. Bold text indicates significant *P*-values.

After 8 weeks, the unadjusted mean SBP was 140 mmHg in the intervention group and 142.4 mmHg in the control group. The mean DBP was 83.8 mmHg in the intervention group and 84.2 mmHg in the control group. After 12 months, the mean SBP was 140.6 mmHg in the intervention group and 142.6 mmHg in the control group while the mean DBP was 83.6 mmHg in the intervention group and 84.1 mmHg in the control group.

The BP was less than 130/80 mmHg for 18.6% of the participants in the intervention group after 8 weeks, compared with 13.4% in the control group (*P* = 0.034). After 12 months, 14.5% in the intervention group and 14.0% in the control group had a BP of less than 130/80 mmHg (*P* = 0.856).

### Antihypertensive medication

The average number of antihypertensive drugs was similar in both the intervention and the control group at all three measuring points (Table 3). The number of participants differs from Table 2 because of missing data on antihypertensive drugs. In both groups, the number of drugs increased significantly from baseline to 12 months, in the intervention group from 1.65 to 1.73 (*P* < 0.001) and in the control group from 1.60 to 1.71 (*P* < 0.001). There was no statistically significant difference in the increase between the two groups (*P* = 0.614).

**TABLE 3:** Proportion of participants with number of antihypertensive drugs

	Baseline		8 weeks		12 months	
No. drugs	Int, *n* (%) Total *n* = 432	Con, *n* (%) Total *n* = 417	Int, *n* (%) Total *n* = 420	Con, *n* (%) Total *n* = 405	Int, *n* (%) Total *n* = 409	Con, *n* (%) Total *n* = 386
1	216 (50.0)	217 (52.0)	195 (46.4)	195 (48.1)	182 (44.5)	176 (45.6)
2	158 (36.6)	159 (38.1)	169 (40.2)	167 (41.2)	159 (38.9)	150 (38.9)
3	51 (11.8)	32 (7.7)	48 (11.4)	34 (8.4)	55 (13.4)	46 (13.4)
4+	7 (1.6)	9 (2.1)	8 (1.9)	9 (2.2)	11 (2.7)	11 (2.9)

Con, control group; Int, intervention group.

### Motivational messages

At baseline, 402 of 482 participants in the intervention group chose to receive motivational messages during the intervention period. There was no significant difference regarding the proportion of participants with a BP of less than 140/90 mmHg when compared within the intervention group. In all, 50% (*n* = 197/394) of the participants who chose to receive motivational messages had a BP of less than 140/90 mmHg after 8 weeks compared with 42% (*n* = 29/69) of those in the intervention group who chose not to receive messages (*P* = 0.222). After 12 months, 47.4% (*n* = 179/378) and 45.3% (*n* = 29/64) had a BP of less than 140/90 mmHg.

When excluding the participants who chose not to receive motivational messages and comparing them to the control group, there was a significant difference between the groups at 8 weeks and a close to significant difference after 12 months (Table 4).

**TABLE 4:** Comparison of participants in the intervention group who chose to receive motivational messages with the control group

	Visit	Intervention, with motivational messages [*n* (%)]	Control [*n* (%)]	*P* value
	Baseline	143/402 (35.6)	165/467 (35.3)	0.941
BP <140/90 mmHg	8 weeks	197/394 (50.0)	181/454 (39.9)	**0.003**
	12 months	179/378 (47.4)	172/420 (41.0)	0.069

## DISCUSSION

### Principal findings

An interactive web-based self-management system using the patient's mobile phone was tested and compared with usual care for the management of hypertension in a primary care setting. After 8 weeks, there was a significant increase in the proportion of participants with a BP of less than 140/90 mmHg in the intervention group compared with the control group. Almost half of the participants in the intervention group had a BP of less than 140/90 mmHg after 8 weeks and 12 months – compared with one-third at baseline. However, after 12 months, the difference between the intervention and the control group had decreased somewhat and was not statistically significant any longer.

### Comparisons to previous work

The increase seen in the proportion of participants with a BP of less than 140/90 mmHg after 8 weeks indicates that an e-health intervention, such as the system used in this study, can be utilized in BP management in primary care. This adds to the evidence of the important role that e-health interventions can play in hypertension management [3,24].

The improvement in the number of participants with a BP of less than 140/90 mmHg seen after 8 weeks and after 12 months was not substantial; more than half of the patients still had uncontrolled BP. The improvement is not very large but since hypertension is such an important cardiovascular risk factor, every step in the right direction is important. The result of this study reflects the difficulty to reach target BP levels. Future analysis may bring clarity to which patients are most likely to benefit from a digital intervention such as this one.

The system addresses several barriers to the successful treatment of hypertension, with a primary focus on strengthening the patient's ability to self-manage the condition. It can be used by prescribing physicians to titrate antihypertensive treatment. Home BP monitoring is recommended over office BP monitoring for treatment titration as it is associated with better BP control [12,25]. In a recent publication, McManus *et al.* described a positive effect on BP control by using a digital intervention for hypertension management, which included self-monitoring, behavioural support, and treatment titration. They concluded that the effect was because of increased titration of antihypertensive drugs [26]. In this trial, the increase in the number of drugs was the same in the intervention and the control group, hence, this is probably not the explanation for the positive effect seen in this trial. More likely, the effect is caused by improved patient adherence to treatment, both medication and lifestyle habits, which in turn is because of participants’ increased insight and motivation. Ongoing analyses of included questionnaires in our study will tell if the participants’ preferences, self-efficacy and beliefs about hypertension and medication have been affected.

In previous studies, it has been concluded that self-monitoring of BP is effective in lowering BP when combined with counselling or education [4] In this study, it would have been an option to include a third arm, were participants received a BP monitor but no other intervention and compared that arm to the intervention group and the control group.

There was a small decrease in the proportion of participants with a BP of less than 140/90 mmHg in the intervention group after 12 months compared with after 8 weeks; 47.1% compared with 48.8%. Lifestyle changes are known to be difficult to maintain over time [27] and some decline in the result would be expected. It is possible that repeating the intervention at regular intervals would bring better results over time. Furthermore, the duration of the intervention may also affect the result. It was noted in the pilot study that the effect on BP levelled off after using the system for 8 weeks and no additional effect was seen when using the system for a longer time period [14].

At 12 months, the intervention group still had a higher proportion achieving the primary outcome (47.1 versus 41.0%) but this difference was no longer statistically significant. Agreeing to partake in a hypertension trial may positively influence the participants’ lifestyle choices and motivation, even if randomized to the control group. Another possible explanation is that participants in the control group were indirectly affected by the intervention since the same participating healthcare professionals met with patients from both the intervention and the control group during the study. In a qualitative study with focus group interviews with participants in PERHIT, healthcare professionals reported that they deepened their understanding of hypertension management and found new ways to talk about BP when using the system [7]. Thus, it is likely that this also affected the patients in the control group and could also explain the increase in fraction achieving the BP target from baseline to 8 weeks (35.3–39.9%). Another aspect supporting this is that antihypertensive drug use increased significantly in the control group. To avoid this form of contagious bias, randomization would have to be on a PHCC level instead, but that would bring risks of other biases, such as socioeconomic differences.

According to the ESC/ESH guidelines from 2018 [2], treated BP values should be targeted to 130/80 mmHg or lower in most patients if tolerated. There are no updated national guidelines in Sweden and less than 140/90 mmHg as a target is still used in clinical practice. This may be the explanation for the low number of participants in this study reaching a BP of less than 130/80 mmHg. There was a significant difference between the groups after 8 weeks (18.6% in the intervention group versus 13.4% in the control group) but after 12 months, the difference was not significant.

In the pilot study, where the same system was tested in a smaller setting, the observed effect was larger than in this study [14]. There are many possible explanations for this. In a large study like PERHIT, the research group has less control over how the intervention is utilized at the different study centres during the study period. In the qualitative study from PERHIT mentioned before [7], it was observed that the system was not used as intended in some cases. For example, some of the patients and healthcare professionals were not aware of (or chose to ignore) the possibility to acquire graphical feedback of reported values, thereby missing an important possibility for increased insights concerning the interplay between lifestyle and BP.

The opportunity to receive motivational messages was declined by 17% of the participants in the intervention group. Our results indicate that BP control was positively affected by receiving motivational messages after 8 weeks of intervention but that the effect waned over time. Furthermore, it was no longer statistically significant after 10 additional months without intervention. Previous literature supports using text messages in hypertension management and a recent meta-analysis found that health education via one-way text messages can be effective in improving BP control [28]. From our results, it cannot be concluded if motivational messages on their own are effective in lowering BP.

### Strengths and limitations

This study was conducted in primary care settings, which makes the results highly relevant for real-life hypertension management, as the vast majority of patients with hypertension are treated in primary care in Sweden. It was a large study, including patients and professionals from different regions and socioeconomic areas. By following the patients for 1 year, the long-term effects could be studied.

There is a risk of recruitment bias in a study like this. The patients may have chosen to participate as they already have an interest in health-related issues and a motivation to change their lifestyle. Even though efforts were made to include patients regardless of previous technical knowledge and experience, there is a risk that patients who are unaccustomed to technical systems or using mobile phones declined to participate. More than 95% of the participants originated from Sweden, thus we are unsure if the results are applicable in a more heterogenic population. This may be because of the inclusion criterion of sufficient understanding of the Swedish language, which was necessary to utilize the system at present. If the system becomes available in other languages, more patients may be able to use it.

In conclusion, the proportion of participants with a controlled BP of less than 140/90 mmHg increased after using the interactive system for self-management of hypertension for 8 weeks, compared with usual care. The trend continued after 12 months, although the difference between the groups decreased and was no longer significant. The results indicate that the effect of the intervention is significant, but the long-term effect is uncertain.

## ACKNOWLEDGEMENTS

We are indebted to Patrick O’Reilly for his professional English language editing assistance, Jenny Sandgren for her professional assistance with statistical analyses and Gun Skire for support with the introduction of the web-based system to the PHCCs.

Source of funding: this work was supported by the Kamprad Foundation under Grant 20170102, the Heart- and Lung Foundation under Grant 20170251 and Grant 20200507, the Swedish Research Council under Grant 2018-02648, and Gothenburg University Centre for Person-Centred Care (GPCC).

### Conflicts of interest

There are no conflicts of interest.

## Supplementary Material

Supplemental Digital Content
