# Supplementary material for: PERson-centredness in Hypertension management using Information Technology: a randomized controlled trial in primary care
Source: J Hypertens. 2022 Nov 18;41(2):246–53. doi: 10.1097/HJH.0000000000003322 (PMC9799039; doi:10.1097/HJH.0000000000003322)
Supplement: Supplemental Digital Content [file jhype-41-246-s001.docx]

**Supplementary table.** Baseline characteristics of participants excluded for any reason during the study.

| **Excluded before 8 weeks** | | | |
| --- | --- | --- | --- |
|  | **Intervention n=19** | **Control n=13** | **p-value** |
| Age (years), mean (SD, range) | 67.4 (10.8, 33-80) | 56.3 (12.0, 33-75) | 0.010 |
| SBP (mmHg), mean (SD) | 146.4 (20.1) | 141.9 (20.3) | 0.533 |
| DBP (mmHg), mean (SD) | 82.5 (13.3) | 86.1 (8.7) | 0.406 |
| Sex, women (%) | 11 (57.9) | 9 (69.2) | 0.515 |
| **Excluded between 8 weeks and 12 months** | | | |
|  | **Intervention n=21** | **Control n=34** | **p-value** |
| Age (years), mean (SD, range) | 63.2 (13.2, 25-78) | 68.0 (10.0, 45-83) | 0.137 |
| SBP (mmHg), mean (SD) | 139.9 (21.8) | 148.4 (18.7) | 0.131 |
| DBP (mmHg), mean (SD) | 83.1 (8.5) | 85.9 (11.8) | 0.347 |
| Sex, women (%) | 9 (42.9) | 14 (41.2) | 0.902 |
| **Excluded total** | | | |
|  | **Intervention n=40** | **Control n=47** | **p-value** |
| Age (years), mean (SD, range) | 65.2 (12.1, 25-80) | 64.7 (11.7, 33-83) | 0.480 |
| SBP (mmHg), mean (SD) | 143.0 (21.0) | 146.6 (19.1) | 0.408 |
| DBP (mmHg), mean (SD) | 82.8 (10.9) | 86.0 (11.0) | 0.187 |
| Sex, women (%) | 20 (50.0) | 23 (48.9) | 0.921 |
